# Relationship between Altered miRNA Expression and DNA Methylation of the DLK1-DIO3 Region in Azacitidine-Treated Patients with Myelodysplastic Syndromes and Acute Myeloid Leukemia with Myelodysplasia-Related Changes

**DOI:** 10.3390/cells7090138

**Published:** 2018-09-14

**Authors:** Michaela Dostalova Merkerova, Hana Remesova, Zdenek Krejcik, Nikoleta Loudova, Andrea Hrustincova, Katarina Szikszai, Jaroslav Cermak, Anna Jonasova, Monika Belickova

**Affiliations:** 1 Institute of Hematology and Blood Transfusion, U Nemocnice 1, 128 20 Prague 2, Czech Republic; hana.remesova@uhkt.cz (H.R.); zdenek.krejcik@uhkt.cz (Z.K.); nikoleta.loudova@uhkt.cz (N.L.); andrea.mrhalkova@uhkt.cz (A.H.); katarina.szikszai@uhkt.cz (K.S.); jaroslav.cermak@uhkt.cz (J.C.); monika.belickova@uhkt.cz (M.B.); 2 General University Hospital, 128 08 Prague, Czech Republic; atjonas@hotmail.com

**Keywords:** microRNA, myelodysplastic syndromes, acute myeloid leukemia, azacitidine, 14q32, MEG3

## Abstract

The *DLK1–DIO3* region contains a large miRNA cluster, the overexpression of which has previously been associated with myelodysplastic syndromes (MDS). To reveal whether this overexpression is epigenetically regulated, we performed an integrative analysis of miRNA/mRNA expression and DNA methylation of the regulatory sequences in the region (promoter of the *MEG3* gene) in CD34+ bone marrow cells from the patients with higher-risk MDS and acute myeloid leukemia with myelodysplasia-related changes (AML-MRC), before and during hypomethylating therapy with azacytidine (AZA). Before treatment, 50% of patients showed significant miRNA/mRNA overexpression in conjunction with a diagnosis of AML-MRC. Importantly, increased level of *MEG3* was associated with poor outcome. After AZA treatment, the expression levels were reduced and were closer to those seen in the healthy controls. In half of the patients, we observed significant hypermethylation in a region preceding the *MEG3* gene that negatively correlated with expression. Interestingly, this hypermethylation (when found before treatment) was associated with longer progression-free survival after therapy initiation. However, neither expression nor methylation status were associated with future responsiveness to AZA treatment. In conclusion, we correlated expression and methylation changes in the *DLK1–DIO3* region, and we propose a complex model for regulation of this region in myelodysplasia.

## 1. Introduction

Myelodysplastic syndromes (MDS) represent a heterogeneous spectrum of hematopoietic stem cell (HSC) disorders characterized by inefficient hematopoiesis, peripheral blood cytopenia, and dysplasia in one or more myeloid cell lineages; they also have a tendency to evolve into acute myeloid leukemia with myelodysplasia-related changes (AML-MRC), which develops in approximately 30–40% of patients [1,2]. In recent years, the hypomethylating agent azacitidine (AZA) has become the standard therapy for higher-risk MDS and AML-MRC. AZA prolongs patient survival, improves clinical outcomes and life quality, and delays progression to AML. However, response to AZA therapy occurs in only approximately 40–50% of MDS patients [3,4,5]. AZA is a cytidine analog, which at low doses functions as a DNA methyltransferase inhibitor, causing DNA hypomethylation; at high doses, AZA shows direct cytotoxicity to abnormal bone marrow (BM) hematopoietic cells through its incorporation into DNA and RNA, resulting in cell death [6].

**Figure 1:**
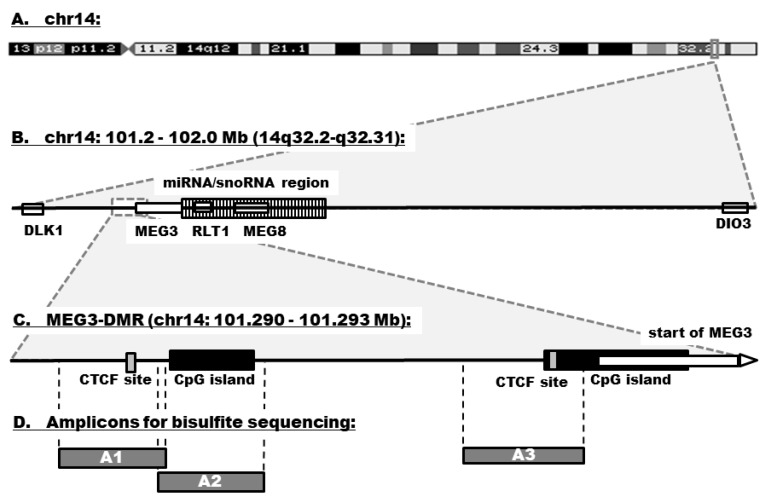
Genomic organization of the *DLK1–DIO3* domain. (**A**) The region is located on chromosome 14q32.2. (**B**) It contains three paternally expressed protein-coding genes (*DLK1, RTL1*, and *DIO3*), three maternally expressed noncoding genes (*MEG3, MEG8,* and antisense *RTL1*), many snoRNAs, and a large cluster of 54 miRNAs (striped bar). (**C**) The expression within the region is under the control of *MEG3*-DMR that overlaps with the promoter and the 5′-end of the *MEG3* gene. *MEG3*-DMR contains several CTFC binding sites (gray bars) and CpG islands (black bars). (**D**) The schema shows localization of amplicons (A1–A3) in the region that were used for methylation analyses.

The *DLK1–DIO3* genomic region is located on chromosome 14q32 and contains three paternally expressed protein-coding genes (*DLK1, RTL1*, and *DIO3*), three maternally expressed noncoding genes (*MEG3, MEG8,* and antisense *RTL1*), many small nucleolar RNAs (snoRNAs), and one of the largest miRNA clusters (54 miRNAs) in the human genome [7] (Figure 1). Expression within the *DLK1–DIO3* region is under the control of three differentially methylated regions (DMRs): Intergenic DMR (IG-DMR), *DLK1*-DMR, and *MEG3*-DMR [8]. The *MEG3-*DMR overlaps with the promoter and the 5′-end of the *MEG3* gene and includes several binding sites for CCCTC-binding factor (CTCF). CTCF blocks the interaction between enhancers and promoters, causing transcriptional repression. It exerts its regulatory function by binding to unmethylated DNA, thus preventing the expression of target genes [9].

The miRNAs in the *DLK1–DIO3* region possess oncogenic and tumor suppressor properties and are frequently deregulated in various cancers [7]. Their deregulation has been linked to abnormal induction of apoptosis and suppression of proliferation [10,11,12]. They are also involved in hematopoietic stem/progenitor cell (HSPC) differentiation [13,14,15]. The upregulation of miRNAs in the *DLK1–DIO3* region has been reported in acute promyelocytic leukemia (APL, a subtype of AML with t(15;17)) [16,17,18,19] and MDS [20,21,22,23]. In APL, a comparison of diagnostic and remission patient samples showed that strong upregulation of these miRNAs was correlated with hypermethylation at *MEG3-*DMR, including the CTCF binding site motifs [24]. Only one publication has studied the methylation of this region in MDS patients; Benetatos et al. (2010) showed that hypermethylation of the *MEG3* promoter occurred in 35% of MDS patients [25].

Using SNP array-based karyotyping in cytogenetically normal MDS patients, we previously demonstrated that upregulation of the miRNAs located in the *DLK1–DIO3* domain seen in MDS is probably not caused by any chromosomal aberration or uniparental disomy [22,26]. Due to the similarity between MDS and AML, it may be expected that overexpression of these miRNAs is associated with aberrant hypermethylation of the locus. In this study, we performed a thorough, integrative analysis investigating the expression of miRNAs and mRNAs encoded in the *DLK1–DIO3* region and compared these data with the methylation status of *MEG3*-DMR in CD34+ BM cells (i.e., undifferentiated hematopoietic stem/progenitor cells (HSPCs), including blasts) from higher-risk MDS/AML-MRC patients, particularly focusing on the effects of hypomethylating AZA therapy.

## 2. Materials and Methods

### 2.1. Patients

BM samples from 12 patients with higher-risk MDS (N = 7) or AML-MRC (N = 5) without previous malignancy, chemotherapy, or transplantation were obtained between the years 2010–2013 from the First Department of Medicine, Department of Hematology, General University Hospital, and from the Institute of Hematology and Blood Transfusion in Prague, Czech Rep. Paired samples (N = 24) before and during AZA therapy (collected at the time of the best response, between cycles 4 and 11) were collected from each patient. Patient age ranged from 63–82 years (average 69), and the female/male distribution was 6/6. AZA was administered at 75 mg/m^2^/day for 7 consecutive days every 28 days. The hematological evaluation of the response to treatment was performed according to the International Working Group (IWG) criteria for MDS and AML [27,28]. Seven patients were considered as responders, and five patients were nonresponders. The detailed clinical characteristics of all patients are summarized in Table S1. All samples from this cohort were used for parallel investigation of miRNA/mRNA expression and DNA methylation. BM samples obtained from age-matched healthy donors with no adverse medical histories were used as controls. Due to limited amounts of sample, we had to use different controls for each type of analysis. Written informed consent was obtained from all test subjects in accordance with the approval of the Institutional Review Board (No. NS9634-4/2008).

### 2.2. Cell Separation and Nucleic Acid Extraction

Mononuclear cells (MNCs) were separated from BM aspirates by Ficoll-Paque density gradient centrifugation (GE Healthcare, Munich, Germany). CD34+ cells were isolated from MNCs by the Direct CD34 Progenitor Cell Isolation MACS Kit (Miltenyi Biotec, Bergisch Gladbach, Germany). Total RNA was extracted from separated CD34+ cells using the acid guanidinium-thiocyanate-phenol-chloroform method, and the RNA samples were incubated with DNase I (Qiagen, Hilden, Germany) to prevent genomic DNA contamination. The salting-out method was used to isolate DNA from CD34+ cells. The concentrations of DNA and RNA were assessed using a NanoDrop and a Qubit 2.0 Fluorometer (both from Thermo Fisher Scientific, Waltham, MA, USA). The integrity of total RNA was evaluated by an Agilent Bioanalyzer 2100 (Agilent Technologies, Santa Clara, CA, USA).

### 2.3. miRNA Expression

The miRNA expression data were retrieved from our previous project, which studied miRNA expression changes on a genome-wide level before and during AZA therapy using Agilent Human miRNA Microarrays, Release 19.0, 8 × 60K (Agilent Technologies, Santa Clara, CA, USA) [29]. That study included data on miRNA expression from a complete cohort of 12 patients and four controls. For this publication, we have focused on only miRNAs located in the *DLK1–DIO3* region.

### 2.4. Gene Expression

Data (NCBI GEO accession number GSE77750) from our previous genome-wide analysis performed using Illumina microarrays (HumanHT-12 v4 Expression, Illumina, San Diego, CA, USA) were used [30]. For this project, the expression of only five genes in the *DLK1–DIO3* region (*DLK1, RTL1*, *DIO3, MEG3,* and *MEG8*) and of the *CTCF* was further considered within our patient cohort (the same 12 patients with MDS/AML-MRC) and 10 controls.

For validation, the expression of *MEG3* was measured in an independent cohort of samples using RT-qPCR. The cohort included 79 patients with primary MDS (12 MDS with del(5q), 8 MDS with single lineage dysplasia (SLD), 8 MDS with ring sideroblasts and SLD (RS-SLD), 12 MDS with multilineage dysplasia (MLD), 8 MDS-RS-MLD, 10 MDS with excess blasts 1 (EB1), 21 MDS-EB2), 12 AML-MRC, and 13 age-matched healthy controls. SuperScript IV VILO Master Mix and the TaqMan Gene Expression Assay with TaqMan Universal Master Mix (all from Thermo Fisher Scientific, Waltham, MA, USA) were used. The data were normalized to *HTFR* as the reference gene and processed with the ddCt method.

### 2.5. Bisulfite Sequencing

Genomic DNA isolated from CD34+ BM cells from the 24 samples (the same set of paired samples from 12 patients as in the expression analyses) and three healthy controls was used for analysis of the methylation status of *MEG3*-DMR. Bisulfite conversion was performed using EpiTect Bisulfite Kit (Qiagen, Hilden, Germany). Based on the publication by Manodoro et al. [24], three regions with significant methylation changes in APL were chosen, and previously published primers for these amplicons (A1, A2, and A3) were used (Table S2). Amplicons were generated using FastStart High Fidelity PCR System (Roche Applied Science, Mannheim, Germany) and purified with Agencourt AMPure beads (Beckman Coulter, Krefeld, Germany). The purified PCR products were sequenced using the Roche 454 GS Junior System. Image processing was performed the GS RunBrowser software (Roche Applied Science). Methylation status of CpG sites was quantified using QUMA web-based tool [31].

### 2.6. Statistical Analyses

Statistical analyses were performed using the GraphPad Prism v7.03 (GraphPad Prism Software, La Jolla, CA, USA). Unpaired and paired t-tests were used to compare miRNA/mRNA expression levels and clinical parameters between different groups of samples. The chi-square test was applied for comparison of categorical clinical variables. Hierarchical clustering analysis was done using average linkage and Euclidean distance. Correlation analyses were done by computing Pearson correlation coefficients (r). Overall survival (OS) and progression-free survival (PFS) curves were generated by the Kaplan-Meier method, and the differences between groups were assessed by the log-rank test. OS and PFS were defined as the time from the beginning of treatment until death from any cause (OS) or disease progression (PFS). Differences were considered statistically significant at *p* < 0.05.

## 3. Results

### 3.1. Expression of miRNAs Encoded within the DLK1–DIO3 Region

**Figure 2:**
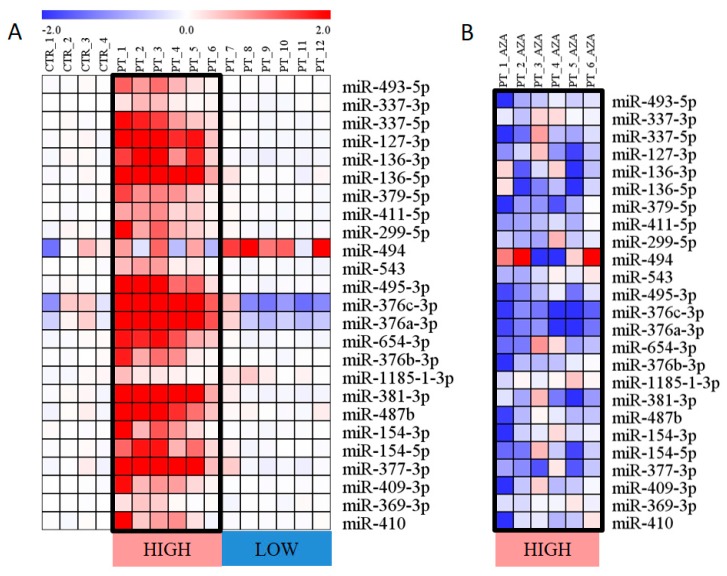
Expression of miRNAs encoded in the *DLK1–DIO3* region. (**A**) miRNA expression in untreated patients (PT) and healthy controls (CTR). The expression level is calculated as the binary logarithm of fold change (logFC) compared to the mean expression of controls. The miRNAs are aligned in the heatmap according to their location on chr14. (**B**) Reduction of miRNA expression following AZA treatment. The changes in miRNA levels are calculated as the logFC between paired samples (AZA treatment vs. pretreatment). Only those patients with miRNA overexpression before treatment are shown. Both heatmaps use a color gradient intensity scale to express visually the logFC values in a range of colors (blue—downregulation, red—upregulation, white—unchanged expression).

Within the microarray miRNA expression data, signals of 25 miRNAs located in the *DLK1–DIO3* region were detected. Their levels varied by three orders of magnitude in microarray signal intensity. Before treatment, increased expression of these 25 miRNAs was detected in 6 of 12 samples (50% of patients), and in the remaining 6 samples, the miRNA expression was similar to that detected in controls (Figure 2A).

Furthermore, we compared miRNA expression as paired samples from pretreatment and during AZA therapy. miRNA expression was reduced following therapy specifically in the patients with overexpressed miRNAs before treatment, and their levels decreased to approximately half the pretreatment expression levels. In contrast, the levels of these miRNAs remained unchanged in the patients with baseline miRNA levels before AZA treatment (Figure 2B).

### 3.2. Expression of Genes Related to the DLK1–DIO3 Region

**Figure 3:**
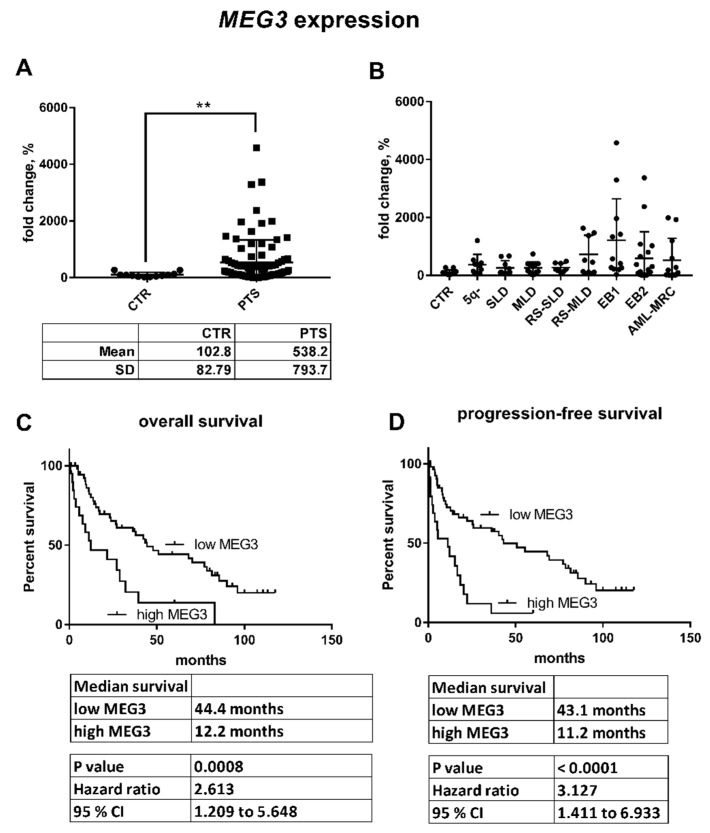
Relative *MEG3* expression in the validation cohort of MDS/AML-MRC patients. (**A**) The expression of *MEG3* was significantly different between healthy controls (CTR) and all patients (PTS) (** *p* < 0.01). (**B**) Further distribution of the patients according to their diagnosis revealed increased expression of *MEG3* particularly in the patients with advanced disease. (**C**) Overall survival and (**D**) progression-free survival of patients with low vs. high *MEG3* expression (the cut-off was set up to mean level of *MEG3*).

Expression of five genes encoded in the *DLK1–DIO3* region (*DLK1, RTL1*, *DIO3, MEG3,* and *MEG8*) and of the *CTCF* gene were measured using microarrays in the same cohort of patients as for the miRNA analysis. Signals of probes for the *RTL1* and *DIO3* genes were below the detection limit. Expression of *DLK1*, *MEG3*, and *MEG8* followed the same trend as that of the clustered miRNAs; their levels were increased in the untreated patients with elevated miRNA expression and subsequently reduced during AZA therapy. In the patients with baseline miRNA expression, the transcript levels remained unchanged compared with the levels seen in controls. Interestingly, expression of the *CTCF* gene was also moderately higher (*p* = 0.185) in the patients with increased miRNA expression in the *DLK1–DIO3* region and then reversed to baseline after therapy (*p* = 0.026) (Figure S1). Quantification of *MEG3* expression in a larger, independent cohort of patients showed significant upregulation (*p* = 0.0049) of *MEG3* in MDS/AML-MRC patients compared to healthy donors (Figure 3A). Interestingly, this upregulation occurred more frequently in patients with higher-risk diagnoses than in those with early subtypes of MDS (Figure 3B) and was associated with poor OS (hazard ratio [HR] = 2.6013, 95% confidence interval [CI] = 1.209 to 5.648, *p* = 0.0008) and PFS (HR = 3.127, 95% CI = 1.411 to 6.933, *p* < 0.0001) in untreated patients (Figure 3C,D).

### 3.3. DNA Methylation in the MEG3-DMR

For DNA methylation analysis, amplicon libraries from three distinct regions overlapping the *MEG3*-DMR were prepared, and a total of 53,849 read sequences were generated using amplicon bisulfite sequencing. After a quality control check, 471 sequence reads on average were analyzed per amplicon per sample. To validate the reproducibility of the sequencing, we compared the methylation level of two overlapping CpGs independently sequenced in amplicons A1 and A2, which proved to have a high level of correlation (r = 0.901, *p* < 0.001).

**Figure 4:**
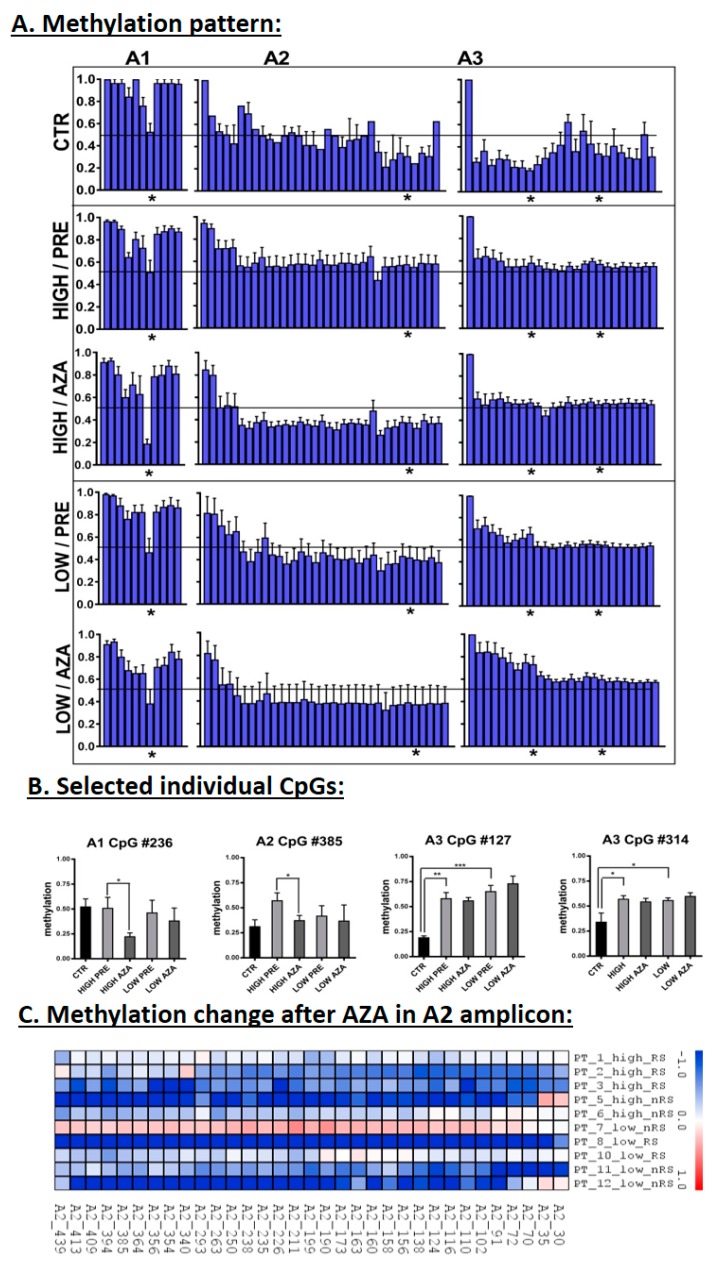
DNA methylation of the *MEG3*-DMR. (**A**) Average methylation profiles examined in the A1–A3 amplicons. Samples were separated into one control group (CTR) and four patient groups that were then divided according to the miRNA expression (HIGH or LOW groups) and AZA therapy (PRE—pretreatment, AZA—AZA-treatment). The horizontal line spanning the plots indicates 50% of methylation. (**B**) Methylation of individual CpGs that are marked in the first section by stars below the graphs. * *p* < 0.05, ** *p* < 0.01, *** *p* < 0.001. (**C**) Methylation change in the A2 amplicon after AZA treatment. Methylation change is expressed as the logFC in AZA-treated samples compared with paired pretreatment samples using a color gradient intensity scale (blue—hypomethylation, red—hypermethylation, white—unchanged methylation). RS—responders to AZA, nRS—nonresponders to AZA.

Average methylation profiles examined throughout all three amplicons revealed several differentially methylated CpGs among various groups of samples (Figure 4A). The comparison between patients and controls showed significant hypermethylation of almost all CpGs in the A3 region in patients (overall hypermethylation >30% compared with the mean of the controls, which was observed in 50% of untreated patients) (Figure 4A,B). Following AZA therapy, a majority of CpGs from the A2 region (Figure 4C) and one CpG (#236) from the A1 region (Figure 4B) were significantly hypomethylated compared with the pretreatment status, particularly in the patients with increased miRNA levels. Finally, we examined methylation of CpGs in two CTCF binding sites located in our amplicons (CTCF_B: Amplicon A1 CpGs #311-319, and CTCF_D: Amplicon A3 CpGs #308-314) and detected significant hypermethylation of A3 CpG #314 in patient samples (Figure 4B).

### 3.4. Correlation of Expression and Methylation Data

We performed a series of pairwise correlation analyses of the expression of selected miRNAs/mRNAs across all patient data (Table S3). Based on this analysis, we identified strong correlation of expression levels of majority of clustered miRNAs. Moreover, the level of *MEG3* positively correlated with the levels of *MEG8, DLK1,* and the miRNAs. However, *CTCF* expression was not significantly correlated with the levels of any of the tested genes/miRNAs.

Pairwise correlation analyses were performed also for DNA methylation data (Table S3). A significant positive correlation of the methylation levels of individual CpGs located nearby was detected. Four separate clusters regulated in common were identified: CpGs from the A1 amplicon, those from the A2 amplicon, and two separate CpG clusters within the A3 amplicon (A3_1—CpGs #54-171 and A3_2—CpGs #227-388).

Importantly, the correlation of methylation status of individual CpGs with miRNA/mRNA expression levels identified that methylation in a part of the A3 region (CpGs #54–231) is negatively correlated with the expression of the majority of the studied miRNAs/mRNAs, with the exception of miR-136-3p.

### 3.5. Clinical Characteristics with Relation to the Expression and Methylation Data

Because only a half of the patients showed overexpression of miRNAs/mRNAs from *DLK1–DIO3* region, we divided the cohort according to the expression activity in the locus and analyzed the two resulting groups of patients according to clinical features (age, sex, karyotype, BM blasts, diagnosis, risk category using criteria of the International Prognostic Scoring System (IPSS), and response to hypomethylating therapy) (Table 1). Of these characteristics, only the percentage of BM blasts (patients with low expression: 10.4 ± 2.8%, patients with high expression: 31.6 ± 9.0%; *p* = 0.048) and diagnosis (patients with low expression: 6 MDS patients, patients with high expression: 1 MDS and 5 AML-MRC patients; *p* = 0.015) were significantly associated with expression activity. Concerning cytogenetics, neither particular aberrations nor their classification based on IPSS showed a correlation with expression levels. The rest of the variables, including overall response rate (ORR) to AZA (*p* = 0.558), were not statistically significant.

**Figure 5:**
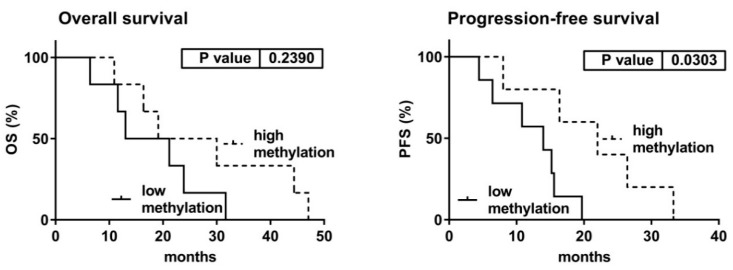
Patient survival after the initiation of AZA therapy according to DNA methylation of the A3 amplicon before treatment. DNA methylation in each sample was considered low or high according to the median value of overall methylation in the locus.

Further, we investigated the survival of patients after the initiation of AZA therapy in relation to miRNA expression and DNA methylation in pretreatment samples. Concerning miRNA levels, we observed no differences in either OS (*p* = 0.901) or PFS (*p* = 0.611) between the patient with baseline vs. increased expression activity (Figure S2). For analysis of DNA methylation, we compared survival of the patients divided according to their overall methylation status in the three examined amplicons. Importantly, we found that PFS (but not OS) significantly correlated with methylation status of the A3 locus. Log-rank test showed that patients with higher methylation of this locus had a significantly longer PFS than those with lower methylation (HR = 0.220, 95% CI = 0.056 – 0.865, *p* = 0.030) (Figure 5).

Finally, we investigated the potential application of the altered DNA methylation of the *MEG3*-DMR for prognostic purposes regarding the responsiveness to AZA treatment. However, we did not find any association between AZA response and methylation level in the analyzed amplicons. Although we observed a substantial decrease in DNA methylation in the A2 region following AZA therapy (Figure 4C), this change occurred in the majority of the patients regardless of their response status.

## 4. Discussion

In several recent publications, apparent overexpression of miRNAs located within the *DLK1–DIO3* region has been observed in MDS patients [20,21,22,23]. Following lenalidomide treatment, higher expression levels of these miRNAs diminished in the patients with low and intermediate-1 risk with a del(5q) aberration [22]. Overexpression of these miRNAs has been linked to hypermethylation of the *MEG3*-DMR in APL [24]. Therefore, we investigated the expression activity within the *DLK1–DIO3* region in the context of the methylation status of the *MEG3*-DMR in myelodysplasia. We focused on patients with higher-risk MDS/AML-MRC diagnoses treated with the hypomethylating agent AZA and examined gene expression levels and DNA methylation status in this chromosomal region following treatment. Although performed on a limited number of patients, our study provides a unique dataset focused particularly on the *DLK1–DIO3* region and includes information on miRNA and mRNA expression and methylation data. Furthermore, all the data were obtained from the same cohort of patients before and during hypomethylation therapy.

Initially, we studied expression within the *DLK1–DIO3* region. Our data confirmed that expression activity throughout the region was regulated uniformly because levels of the miRNAs and mRNAs significantly correlated within our patient cohort. In the untreated MDS/AML-MRC patients, 50% of samples showed significant overexpression of miRNAs and mRNAs encoded within the region. Other publications described increased expression of *DLK1–DIO3*-related miRNAs, even in somewhat higher proportions (approximately 90%) of MDS/AML-MRC patients [20,21,22,23].

Evaluation of expression data with clinical features showed that expression in the pretreatment samples was related to BM blast count, patient diagnosis, and outcome (OS and PFS). High expression was significantly associated with the diagnosis of AML-MRC and poor outcome, whereas low expression was related to MDS and favorable outcome. Because overexpression of these miRNAs has been linked to a specific cytogenetic aberration [t(15;17)] in primary AML (i.e., APL) [19], we inspected the chromosomes of our patients. However, no chromosomal abnormality or cytogenetic risk category was linked to the expression changes. Therefore, we conclude that the increased expression activity could be related to the process of leukemic transformation. Interestingly, *MEG3* overexpression has also been detected in CD34+ cells from patients with primary myelofibrosis (found in 65% of patients), and this upregulation was related to increased blast counts and higher percentages of circulating CD34+ cells [32].

As half of the patients in our cohort had increased expression activity in the *DLK1–DIO3* region, we examined whether this increase was affected by hypomethylating therapy. Indeed, we observed a reduction in expression to near normal levels after treatment with AZA. To distinguish whether this effect was directly caused by hypomethylation of DNA in the regulatory sequences of the region or if it was an indirect consequence of other changes caused by AZA exposure, we thoroughly examined the methylation pattern in the *MEG3*-DMR.

We investigated DNA methylation in the same regions (located in the *MEG3*-DMR) that were hypermethylated in APL so that we could compare methylation status in APL and myelodysplasia. In APL, the hypermethylation spread over all the three tested amplicons and diminished in remission, whereas we identified a distinct methylation pattern in our cohort of MDS/AML-MRC patients. In untreated patients, we revealed significant hypermethylation solely in the A3 amplicon (that closely precedes the *MEG3* start), whereas the remaining two amplicons had similar levels of methylation as the healthy controls. The hypermethylation in the A3 amplicon was seen in 50% of the MDS/AML-MRC untreated patients. For comparison, Benetatos et al. identified hypermethylation of the *MEG3* promoter in 35% of MDS and 48% of AML patients using conventional methylation-specific PCR [25]. In our patients, methylation of CpGs from the 5′ upstream part of the A3 locus negatively correlated with the expression of genes encoded in the *DLK1–DIO3* region. Interestingly, hypermethylation of the A3 locus before treatment had a positive impact on PFS after AZA therapy initiation.

Various methylation patterns were observed in the remaining amplicons. Of them, the hypomethylation seen in the whole A2 region following AZA therapy was remarkable. Although significant in only the patients with increased miRNA levels, this methylation change was apparent in a majority of patients regardless of expression activity in the *DLK1–DIO3* region. Because of its widespread detection, this hypomethylation might be attributed to the hypomethylating effect of AZA and could therefore influence the deregulated expression of the miRNA cluster seen in a portion of our patients.

In APL, Manodoro et al. documented an atypical pattern of hypermethylation in the *DLK1–DIO3* locus associated with higher expression of the clustered miRNAs that they attributed to the presence of CTCF binding sites [24]. A CTCF insulator binds to several target sites (to their unmethylated forms) in the *MEG3*-DMR, inhibiting transcriptional activity in the region [9]. Here, we detected significant hypermethylation at one of the CTCF binding sites in the A3 amplicon. This hypermethylation occurred in a majority of patients and was not affected by AZA treatment. Interestingly, expression of the *CTCF* gene was higher in the untreated patients with increased miRNA expression, which might interfere with the potential impact of hypermethylation of its binding site in the *MEG3*-DMR. Unfortunately, expression of the CTCF insulator was not considered in the publication studying APL [24]. In summary, aberrant hypermethylation at one of the CTCF binding sites in the region might affect the activity of CTCF during transcriptional regulation of the *DLK1–DIO3* region, but this effect is probably not as large as in the APL study.

Finally, we investigated the potential application of the deregulation observed in the *DLK1–DIO3* locus for prognostic purposes regarding AZA treatment. Although we identified changes in expression and methylation levels before AZA therapy, these changes were not associated with future responsiveness to treatment. Concerning survival analyses, only methylation status of the A3 amplicon could be useful for the prediction of PFS during treatment. Kaplan-Meier curves suggested that A3 hypermethylation before treatment might serve as a potential positive prognostic marker for PFS after AZA initiation. However, since we evaluated only a small number of patients and no mutation experiments in animal models were done, strict interpretation of these data must be done with caution.

Epigenetic changes have frequently been demonstrated in MDS, especially in the subtypes with higher blast counts. Aberrant methylation has been found to correlate with poor prognosis and survival even in early stage patients [7,33]. Expression in the *DLK1–DIO3* region is known to be epigenetically regulated, and both expression and methylation of this locus have been recognized as altered in myelodysplasia. Here, we correlated changes in expression activity with specific methylation changes in its regulatory sequences in higher-risk MDS and AML-MRC patients treated with AZA and thoroughly investigated a complex regulatory mechanism within this region. Based on our data, we assumed that epigenetic alterations affect expression activity in the locus. Some of these changes have also been documented in APL [24]; however, other observations have had distinct features. Although further investigation in larger datasets and mutation experiments in animal models are required, we conclude that expression of miRNAs located in the *DLK1–DIO3* region is regulated, at least in part, in a different manner than in APL. It seems unlikely that the increased expression in this region in myelodysplasia can be simply attributed to an individual alteration in DNA methylation because we observed several changes that may affect the resulting state. Other mechanisms besides hypermethylation of the *MEG3*-DMR, such as the influence of transcription factors, may also play yet unrecognized roles in the regulation of the *DLK1–DIO3* region in myelodysplasia.

## Figures and Tables

**Table 1:** Clinical characteristics of patients and their stratification according to expression activity in the *DLK1–DIO3* locus.

Variable		Patients with Baseline miRNA Expression	Patients with Increased miRNA Expression	*p*-Value
Age (years)	Mean (range)	73 (65–88)	69 (63–81)	0.294
Sex, *n*	Male	4	2	0.248
	Female	2	4	
Karyotype, *n*	Normal	2	1	0.505
	Abnormal	4	5	
Marrow blasts	Mean (range)	10.4 (5.1–20.0)	31.6 (18.0–75.0)	0.048
Diagnosis, *n*	MDS	6	1	0.015
	AML-MRC	0	5	
IPSS, *n*	Intermediate-1/2	3	0	0.182
	High	3	6	
Response to AZA therapy	ORR, *n* (%)	3 (50%)	4 (67%)	0.558

IPSS—International Prognostic Scoring System, ORR—overall response rate.

## Supplementary Materials

The following are available at: http://www.mdpi.com/2073-4409/7/9/138/s1. Figure S1: Expression of genes within the DLK1–DIO3 region, Figure S2: Overall survival (A) and progression-free survival (B) of MDS/AML-MRC patients after the initiation of AZA therapy according to pretreatment levels of 14q32 miRNAs, Table S1: Patient characteristics, Table S2: List of primers used for the generation of amplicons for bisulfite sequencing, Table S3: Matrix showing pairwise correlations between expression of mRNAs/miRNAs and methylation of CpG sites.
