# Supplementary material for: Relationship between Altered miRNA Expression and DNA Methylation of the DLK1-DIO3 Region in Azacitidine-Treated Patients with Myelodysplastic Syndromes and Acute Myeloid Leukemia with Myelodysplasia-Related Changes
Source: Cells. 2018 Sep 14;7(9):138. doi: 10.3390/cells7090138 (PMC6162661; doi:10.3390/cells7090138)

SUPPLEMENTARY MATERIAL

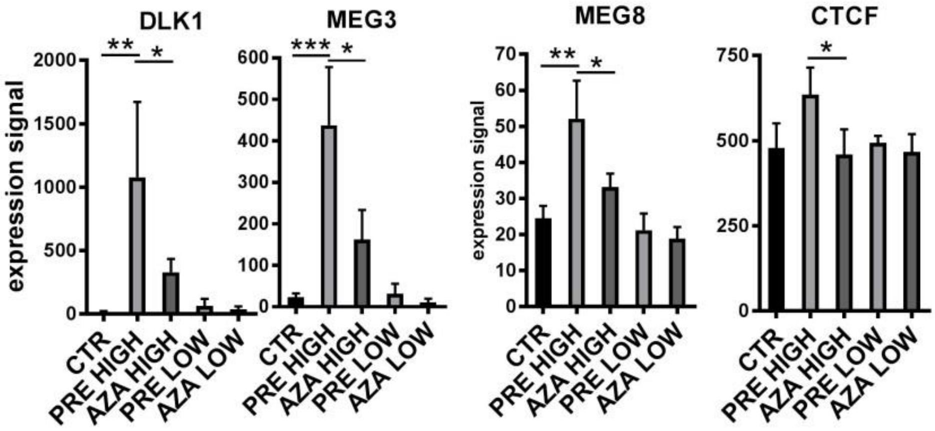

Figure S1. Expression of genes within the *DLK1–DIO3* region. The patient samples were divided according to miRNA expression and treatment status into four groups: PRE HIGH - untreated patients with high miRNA levels, AZA HIGH—AZA-treated patients with high miRNA levels, PRE LOW—untreated patients with low miRNA levels, AZA LOW—AZA-treated patients with low miRNA levels; CTR—controls. *$p < 0.05$, **$p < 0.01$, ***$p < 0.001$.

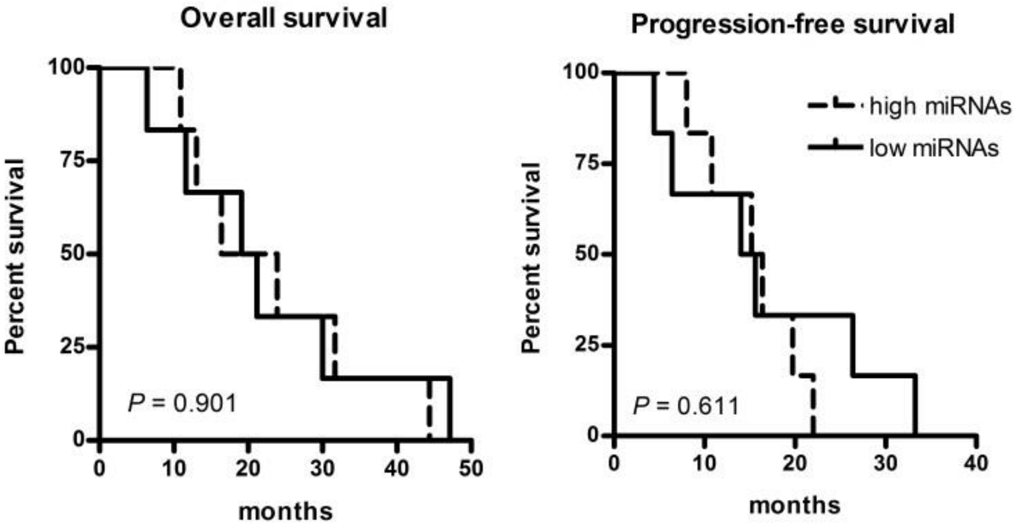

Figure S2. Overall survival (**A**) and progression-free survival (**B**) of MDS/AML-MRC patients after the initiation of AZA therapy according to pretreatment levels of 14q32 miRNAs.

Table S1. Patient characteristics.

**Number of Patients**	**12**
Age (years), mean (range)	69 (63–82)
Sex, female/male	6/6
Marrow blasts (%), mean (range)	18 (4–27)
Karyotype, normal/abnormal	3/9
Diagnosis, MLD/EB1/EB2/AML-MRC	1/1/5/5
IPSS, int-1/int-2/high	1/2/9
AZA cycles administered, median (range)	16 (4–34)
Response, responders/nonresponders	7/5
Follow-up (months), median (range)	25.4 (7.4–94.9)

Table S2. List of primers used for the generation of amplicons for bisulfite sequencing. Positions of amplicons on chr14 were defined according to the publication by Manodoro *et al.* [24] using genome assembly GRCh37/hg19.

Amplicon	Primer	Sequence	Position	Length	Regulatory Elements	CpG Density	Methylation Status in APL [24]
A1	Fwd	AAGTAGGATTGGAAGGTTTTTTAGG	chr14:101,290,069-101,290,515	447	- regulator binding sites: CTCF, OLF-1	0.30	hypermethylation (CpGs 127-421, incl. CTCF binding site)
	Rev	ACAACCCCACAAAAACATAAATTAA					
A2	Fwd	GTATTTTATTTGAGGTTTGGGGTTT	chr14:101,290,455-101,290,946	492	- CpG island<br>- regulator binding sites: NFYA, RFX5, SP1	0.61	hypermethylation (whole region)
	Rev	CACCCCCTTTACAACCTATAAAACT					
A3	Fwd	TATTTTTGGTTAAGGTGGAGGAAG	chr14:101,291,767-101,292,194	428	- CpG island<br>- regulator binding sites: CTCF, RBL2, POLR2A, CREB, PAX3	0.74	hypermethylation (CpGs 25-127 and 278-332, incl. CTCF binding site)
	Rev	CCAAACAAAATAAATTCAAACACAATATAT					

Table S3. Matrix showing pairwise correlations between expression of mRNAs/miRNAs and methylation of CpG sites. Pearson correlation coefficients are provided for each pair of variables. Significant positive correlations are highlighted in a yellow-red scale, and significant negative correlations are shown in a blue scale as indicated at the top of the table. The table is accessible in a separate file at a higher resolution.

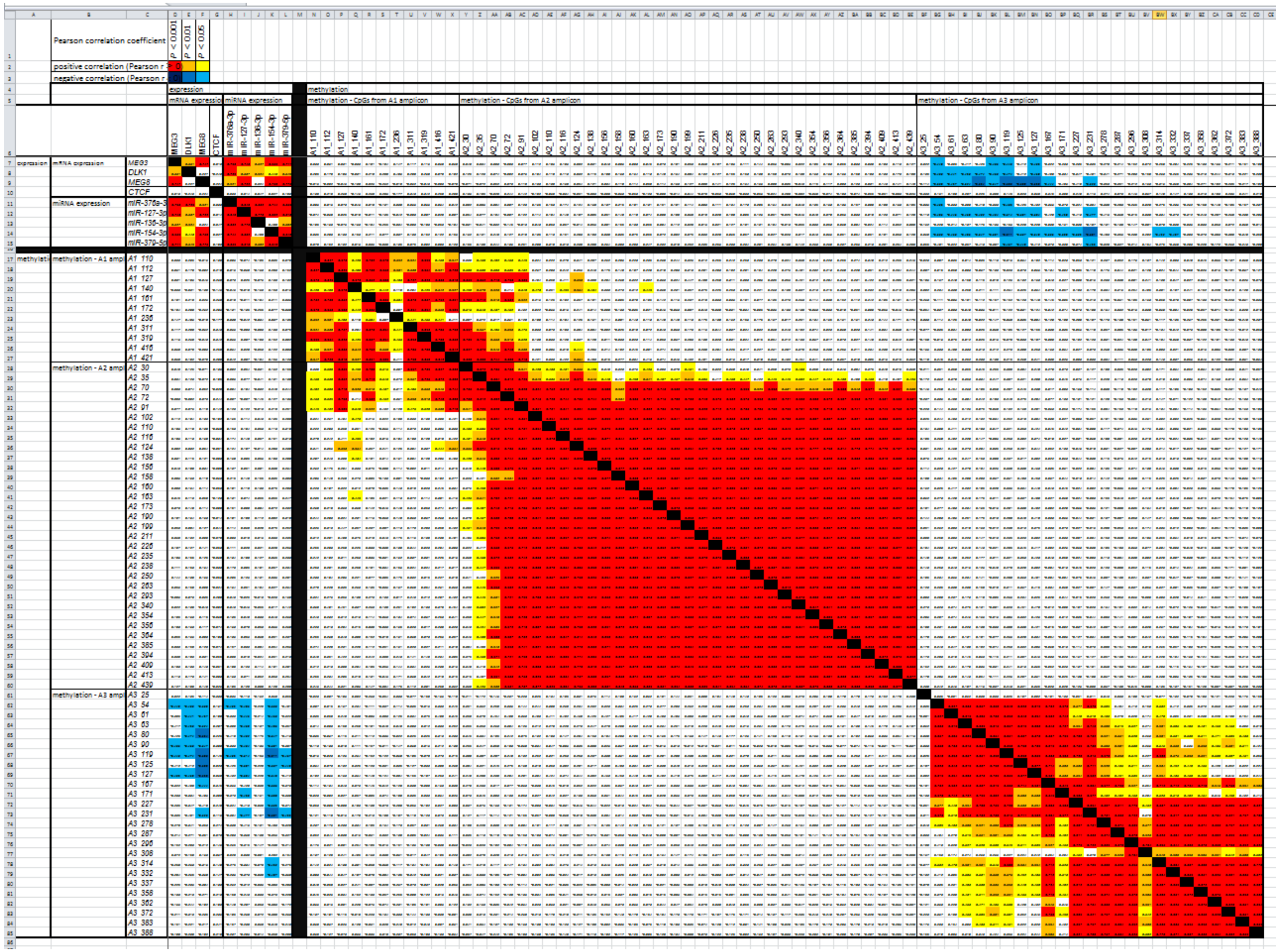

Supplement: Supplementary file 1 [file cells-07-00138-s001.zip › cells-350419-SI/cells-350419-revised 1 (supplementary).pdf]
